# Supplementary material for: Weight-Control Behaviors and Dietary Intake in Chinese Adults: An Analysis of Three National Surveys (2002–2015)
Source: Nutrients. 2023 Mar 14;15(6):1395. doi: 10.3390/nu15061395 (PMC10051790; doi:10.3390/nu15061395)
Supplement: Supplementary file 1 [file nutrients-15-01395-s001.zip › nutrients-2269012-supplementary.pdf]

Supplementary material

Weight-control behaviors and dietary intake in Chinese adults: an analysis of three national surveys (2002–2015)

Miyang Luo [1], Yixu Liu [1], Ping Ye [2], Shuya Cai [3], Zhenzhen Yao [1], Liyun Zhao [3], Jiayou Luo [1,*] and Dongmei Yu [3,*]

[1] Xiangya School of Public Health, Central South University, Changsha 410008, China; miyangluo@csu.edu.cn (M.L.); yixu.liu@csu.edu.cn (Y.L.); y13317437129@163.com (Z.Y.)

[2] Yuxi Center for Disease Control and Prevention, Yuxi 653100, China; yep20227890@163.com (P.Y.)

[3] Key Laboratory of Trace Element Nutrition of National Health Commission, National Institute for Nutrition and Health, Chinese Center for Disease Control and Prevention, Beijing 100050, China; caisy@ninh.chinacdc.cn (S.C.); zhaoly@ninh.chinacdc.cn (L.Z.)

* Correspondence: jiayouluo@126.com (J.L.); yudm@ninh.chinacdc.cn (D.Y.)

Contents

Table S1. CHDI components and criteria for scoring.
Table S2. Characteristics of adults across the three rounds of China National Nutrition Surveys.
Table S3. Univariate analysis of the effect of weight control behavior on diet quality among Chinese adult residents.
Table S4. Logistic regression analysis of the influence of weight control behavior on diet quality among Chinese adult residents.

Table S1. CHDI components and criteria for scoring [1].

CHDI Component	Score Range	Standard for Maximum Score	Standard for Minimum Score of Zero
Food variety	0-10	≥12 kind	≤5 kind
Refined grains	0-5	≥100g/1000kcal	0
Whole grain, dry bean, and tuber	0-5	≥40g/1000kcal	0
Total vegetables	0-5	≥180g/1000kcal	0
Dark green and orange vegetables	0-5	≥90g/1000kcal	0
Fruit	0-10	≥110g/1000kcal	0
Dairy	0-10	≥100g/1000kcal	0
Soybean	0-10	≥10g/1000kcal	0
Meat and egg	0-5	≥50g/1000kcal	0
Fish, shellfish and mollusk	0-5	≥30g/1000kcal	0
Calories from SFAs	0-10	< 10%	≥15%
Sodium	0-10	≤1g/1000kcal	≥4g/1000kcal
Empty calories	0-10	≤20%	≥40%

Abbreviation: CHDI, China Healthy Diet Index; SFAs, saturated fatty acids.

Table S2. Characteristics of adults across the three rounds of China National Nutrition Surveys.

Variable	2002 (*n*=45,148)	2012 (*n*=53,578)	2015 (*n*=68,629)
Gender			
Male	21,670 (50.6)	24,884 (50.9)	32,977 (50.6)
Female	23,478 (49.4)	28,694 (49.1)	35,652 (49.4)
Age group (years)			
18-39	17,400 (43.5)	12,795 (43.8)	13,293 (42.4)
40-64	22,303 (45.1)	30,489 (44.5)	41,259 (47.2)
≥65	5445 (11.4)	10,294 (11.7)	14,077 (10.4)
Education level			
Elementary school and below	18,998 (38.8)	22,180 (33.7)	34,019 (38.9)
Secondary school	23,039 (53.8)	26,864 (54.5)	29,827 (50.2)
College and above	3044 (7.4)	4534 (11.8)	4783 (10.9)
Occupation			
Employed	30,325 (68.0)	34,616 (70.3)	49,681 (76.6)
Unemployed	14,749 (32.0)	18,958 (29.7)	18,948 (23.4)
Annual income per capita			
Low	32,462 (73.4)	26,230 (51.3)	26,066 (44.9)
High	11,791 (26.6)	24,801 (48.7)	31,291 (55.1)
Area			
Urban	15,053 (33.4)	26,561 (48.0)	27,340 (39.5)
Rural	30,095 (66.6)	27,017 (52.0)	41,289 (60.5)
Body mass index (kg/m^2)			
<18.5	2768 (7.3)	2119 (5.6)	2486 (4.7)
18.5-23.9	23,940 (58.1)	23,227 (52.6)	31,325 (49.2)
24-27.9	11,318 (25.7)	15,308 (30.4)	23,421 (32.6)
≥28	3988 (8.9)	5579 (11.4)	9457 (13.5)
Weight control behavior			
Yes	1981 (4.5)	4758 (9.5)	5167 (9.1)
No	43,167 (95.5)	48,820 (90.5)	63,462 (90.9)

Data are *n* (weighted%). Number missing: Occupation (*n*=78), Annual income per capita (*n*=14,714), Education level (*n*=67), Body mass index (*n*=12,419).

Table S3. Univariate analysis of the effect of weight control behavior on diet quality among Chinese adult residents.

Variable	Diet quality: low	Diet quality: high	χ^2	*p*
Overall	134,078 (80.0)	33,277(20.0)		
Gender			28.171	<0.001
Male	64,027 (50.9)	15,504 (49.2)		
Female	70,051 (49.1)	17,773 (50.8)		
Age group (years)			107.212	<0.001
18-39	34,171 (42.6)	9317 (45.5)		
40-64	76,088 (46.4)	17,963 (43.3)		
≥65	23,819 (11.0)	5997 (11.2)		
Education level			7645.021	<0.001
Elementary school and below	66,084 (41.1)	9113 (21.6)		
Secondary school	61,016 (51.4)	18,714 (57.2)		
College and above	6919 (7.5)	5442 (21.2)		
Occupation			978.514	<0.001
Employed	38,821 (26.0)	13,834 (34.7)		
Unemployed	95,194 (74.0)	19,428 (65.3)		
Annual income per capita			4784.859	<0.001
Low	79,370 (64.8)	13,049 (43.0)		
High	42,581 (35.2)	17,641 (57.0)		
Area			10752.582	<0.001
Urban	46,678 (34.3)	22,276 (65.7)		
Rural	87,400 (65.7)	11,001 (34.3)		
Nutrition level			67.366	<0.001
Non-overweight obesity	69,533 (58.9)	16,332 (56.2)		
Overweight obesity	54,290 (41.1)	14,781 (43.8)		
Weight control behavior			1519.822	<0.001
No	126,245 (93.3)	29,204 (86.8)		
Yes	7833 (6.7)	4073 (13.2)		

Data are *n* (weighted%). Number missing: Occupation (*n*=78), Annual income per capita (*n*=14,714), Education level (*n*=67), Nutrition level (*n*=12,419). A China healthy diet index (CHDI) score of ≥60 indicates a high-quality diet, otherwise a low-quality diet. BMI <18.5 kg/m^2 and BMI 18.5-23.9 kg/m^2 were defined as the non-overweight obesity group, and BMI 24-27.9 kg/m^2 and BMI ≥28 kg/m^2 were defined as the overweight obesity group. The univariate analysis was conducted using the Chi-square test.

Table S4. Logistic regression analysis of the influence of weight control behavior on diet quality among Chinese adult residents.

Variable	β	S.E.	Wald	*p*-Value	OR (95% CI)
Gender					
Male					ref
Female	-0.083	0.015	31.711	<0.001	0.920 (0.894,0.947)
Age group (years)					
18-39					ref
40-64	-0.033	0.016	4.114	0.043	0.968 (0.937,0.999)
≥65	-0.138	0.027	26.123	<0.001	0.871 (0.826,0.918)
Education level					
Elementary school and below					ref
Secondary school	-0.529	0.018	835.919	<0.001	0.589 (0.568,0.611)
College and above	-1.010	0.027	1444.971	<0.001	0.364 (0.346,0.384)
Occupation					
Employed					ref
Unemployed	0.241	0.017	210.160	<0.001	1.273 (1.232,1.315)
Annual income per capita					
Low					ref
High	-0.592	0.016	1456.899	<0.001	0.553 (0.537,0.570)
Area					
Urban					ref
Rural	0.849	0.016	2816.688	<0.001	2.337 (2.265,2.412)
Nutrition level					
Non-overweight obesity					ref
Overweight obesity	0.077	0.015	26.635	<0.001	1.080 (1.049,1.113)
Weight control behavior					
Yes					ref
No	0.262	0.024	123.552	<0.001	1.30 (1.241,1.362)

A China healthy diet index (CHDI) score of ≥60 indicates a high-quality diet, otherwise a low-quality diet. BMI <18.5 kg/m² and BMI 18.5-23.9 kg/m² were defined as the non-overweight obesity group, and BMI 24-27.9 kg/m² and BMI ≥28 kg/m² were defined as the overweight obesity group.

References:

1. He, Y.; Fang, Y.; Yang, X. Establishment and application of China healthy diet index. *Acta Nutrimenta Sinica* **2017**, *39*, 436-441. https://doi:10.13325/j.cnki.acta.nutr.sin.2017.05.011.
